# Application of the Occupational Sitting and Physical Activity Questionnaire (OSPAQ) to office based workers

**DOI:** 10.1186/1471-2458-14-762

**Published:** 2014-07-29

**Authors:** Jonine Jancey, Marian Tye, Sarah McGann, Krysten Blackford, Andy H Lee

**Affiliations:** Western Australian Centre for Health Promotion Research, School of Public Health, Curtin University, GPO Box U1987, Perth, 6845 Western Australia; Centre for Sport and Recreation Research, Curtin University, Kent Street, Bentley, Perth 6102 Western Australia; School of Arts and Sciences, University of Notre Dame, Fremantle, 6160 Western Australia; School of Public Health, Curtin University, Kent Street, Bentley, Perth 6102 Western Australia

**Keywords:** Health promotion, Physical activity, Sedentary behaviour, Workplace

## Abstract

**Background:**

The workplace is a setting where sedentary behaviour is highly prevalent. Accurately measuring physical activity and sedentary behaviour is crucial to assess the impact of behavioural change interventions. This study aimed to evaluate the reliability and criterion validity of the Occupational Sitting and Physical Activity Questionnaire (OSPAQ) and compare with data collected by accelerometers.

**Methods:**

A test-retest study was undertaken on 99 participants using the OSPAQ. Data were then compared to accelerometer records of 41 participants. Reliability was assessed by paired t-test and intra-class correlations (ICC) via a two-way mixed model based on absolute agreement. Difference and agreement were measured by comparison of mean self-reported data with accelerometer data using the Pearson’s correlation coefficient and Bland-Altman plots.

**Results:**

The ICCs for minutes spent sitting (0.66), standing (0.83) and walking (0.77) showed moderate to strong test-retest reliability. No significant differences were found between the repeated measurements taken seven days apart. Correlations with the accelerometer readings were moderate. The Bland-Altman plots showed moderate agreement for standing time and walking time but systematic variation for sedentary time.

**Conclusion:**

The OSPAQ appears to have acceptable reliability and validity measurement properties for application in the office workplace setting.

## Background

The workplace is a setting where sedentary behaviour is highly prevalent [1] and where many adults spend the majority of their waking hours [2]. It is now recognised that prolonged sitting at work is an occupational health risk that can have adverse health outcomes for sedentary workers [3], especially those in the office based environment. Sedentary behaviour is associated with adverse health conditions such as obesity, cardiovascular disease, diabetes and cancer [4–8]. This presents an important environment for modifying employee behaviours [9, 10] and a critical setting for the delivery of health promotion interventions designed to increase health enhancing behaviours [11, 12].

Accurately measuring physical activity and sedentary behaviour is crucial in order to assess the impact of health behaviour change interventions [11, 12]. Historically, self-reported measures of physical activity are popular due to their low cost, ease of use, and ability to measure frequency, intensity, duration and type of physical activity [13]. However, increasingly objective measures of physical activity such as pedometers and accelerometers are becoming more common [13], as such devices are able to capture activities that can be difficult to quantify subjectively.

Accelerometers are a form of motion sensor that measure activity intensity by differentiating between low, moderate and vigorous activities [13]. The device is able to read either on one plane (i.e. vertical) or on a multi-axial basis [14], and has been shown to be a sound way for recording sedentary behaviours [15]. The use of accelerometers and the recording of data have been reported on those accelerometers strapped to the thigh, hip and waist [16] with varying success. Strapping to the thigh of particularly sedentary workers in the office environment may make them more sensitive to determine sitting behaviour [1], but its advantage over other locations remains inconclusive.

The present study aims to determine the reliability and criterion validity of the Occupational Sitting and Physical Activity Questionnaire (OSPAQ) [17] for application to office based workers.

## Methods

### Participants

Office based workers were sent an email invitation to participate in the study. The convenience sample of office workers was recruited through the staff directory at a large Australian University. Participants were required to be employed by the University; aged ≥ 18 years; and working full-time in an office-based role. All participants who expressed an interest in the study received a plain language statement and consent form (n = 118) and completed a questionnaire on two occasions (stream one). A subsample of these participants (n = 47) were invited and agreed to wear an accelerometer for five consecutive working days (stream two).

### Procedure

Links to the online questionnaire were sent via email. All participants completed the questionnaire twice seven days apart, while those in stream two wore an accelerometer for five consecutive working days during working hours between occasions one and two. They were instructed to remove the accelerometer before leaving work each day and were sent a reminder email to attach it to their body when they arrived at work each morning. A trained researcher fitted the accelerometer and provided instructions for its use on site. In return for their participation, participants received feedback on their physical activity levels as well as being placed in a draw to win an iPad. The study was approved by the Human Research Ethics Committee of the researchers’ institution (approval number SPH-34-2012).

### Measures

#### Questionnaire

The OSPAQ [12], a questionnaire measuring physical activity and sedentary behaviour, was used. Demographic information including age, gender, educational level, country of birth, and anthropometrics (height and weight) were also collected.

#### Instrument

The OSPAQ is a brief instrument to record the proportion of work time spent sitting, standing, walking, and doing heavy labour, as well as the total length of time worked in the past five working days [12]. It was developed from the MONICA Optional Study of Physical Activity [18] and the Behavioural Risk Factor Surveillance System [19].

#### Accelerometer

Time spent in sedentary, light, moderate, and vigorous activity was objectively measured using an ActiGraph GT3X + accelerometer. Accelerometers were strapped on either the waist or the left thigh. Participants needed to wear the accelerometer for at least 75% of their work day over five consecutive working days to be included in the study [20].

Accelerometer activity counts were recorded in 10-second epochs, downloaded and managed using ActiLife 6 desktop software. Wear time was validated by excluding periods of consecutive strings of zero-count epochs lasting 60 minutes or longer (non-wear time) [17], using no-tolerance and the vertical axis. Freedson cut points were used to compute sedentary, light, moderate, and vigorous activities specific to body location (thigh and hip) [21]. Raw data files were then transformed into excel files before calculating total time spent in each activity type (as labelled by the ActiLife 6 software), as well as the proportion of total work time spent sitting (sedentary activity), standing (light activity), walking (moderate activity), and doing heavy labour (vigorous activity).

### Statistical analyses

Self-reported activity data were calculated by multiplying the percentage of the activity for each domain (sitting, standing, walking) from the OSPAQ by the number of hours worked per day and then converting into minutes [17].

The test-retest reliability of the OSPAQ was first examined using intraclass correlation coefficient (ICC) via a two-way mixed model based on absolute agreement for each domain, whereby items with ICC < 0.4 classified as poor; 0.4-0.75 as fair to good; and ICC > 0.75 as excellent [17]. Paired sample t-test was next used to ascertain the apparent differences between occasion 1 (test) and occasion 2 (retest) for self-reported physical activity. Criterion validity of the questionnaire was assessed by comparing the sitting, standing and walking question responses at occasion two with the accelerometer data (sedentary, light intensity, moderate intensity) using Pearson’s (*r*) correlation coefficients, with *r* < 0.3 considered as weak, 0.30-0.49 as low, 0.50-0.69 as moderate, 0.70-0.89 as strong, and *r* > 0.90 as very strong [17]. All statistical analyses were performed in the SPSS package version 21.

Bland-Altman plots were used to assess whether differences between self-reported and measured data were strongly associated with mean values [22]. For each variable, the plot represents the discrepancies between self-reported and measured values against the mean of self-reported and measured values. Limits of agreement were computed as the mean difference ±1.96 standard deviations, showing the range of discrepancies for 95% of the participants. These plots were generated using the MedCalc version 13.2.2.

## Results

### Participant characteristics

An email invitation was sent to 900 staff members. In total 99 participants who agreed to complete the questionnaire on two occasions (response rate 11%) were invited to participate. Forty-seven of these 99 participants agreed to wear an accelerometer, while 41 (87%) successfully wore an accelerometer on the thigh (n = 19) or waist (n = 22) and contributed to the analysis. Table 1 presents the demographic characteristics of the sample. Most of the participants were female (63.6%) and university educated (78.8%). All participants were able to independently complete the questionnaire within five minutes, and no missing items were reported, confirming operational acceptability of the OSPAQ. Moreover, there were only a few extreme values for sitting, standing and walking for both self-reported and objective measures. The percentage of participants showing the highest and lowest values ranged from 2.5% to 4.9%, so that floor/ceiling effects were not evident.

**Table 1:** **Participant characteristics**

Characteristic			Accelerometer wearers	
		Stream 1 (n = 99)	Stream 2a right thigh (n = 19)	Stream 2b waist (n = 22)
		N (%)	N (%)	N (%)
**Gender**	Male	36 (36.4%)	6 (31.6%)	11 (50.0%)
	Female	63 (63.6%)	13 (68.4%)	11 (50.0%)
**Age**	18-29	27 (27.3%)	4 (21.0%)	10 (45.5%)
	30-39	29 (29.3%)	3 (15.8%)	6 (27.3%)
	40-49	23 (23.2%)	9 (47.4%)	2 (9.1%)
	50+	20 (20.2%)	3 (15.8%)	4 (18.2%)
**Country of birth**	Australia	55 (55.6%)	8 (42.1%)	11 (50.0%)
	Other	44 (44.4%)	11 (57.9%)	11 (50.0%)
**Education level**	Year 10 or less	3 (3.0%)	0 (0.0%)	1 (4.5%)
	Year 12	8 (8.1%)	4 (21.1%)	0 (0.0%)
	TAFE/Diploma	11 (11.1%)	2 (10.5%)	3 (13.6%)
	University degree	77 (77.7%)	13 (68.4%)	18 (81.8%)
**Body mass index (WHO cut points)**	Healthy weight (18.50-24.99)	45 (45.4%)	8 (42.1%)	12 (54.5%)
	Overweight (25.00-29.99)	36 (36.4%)	9 (47.4%)	6 (27.3%)
	Obese (>30.00)	18 (18.2%)	2 (10.5%)	4 (18.2%)

### Test-retest reliability

On average, the participants sat in excess of 1900 minutes over five consecutive days, or almost 6.4 hours per work day. All measures in the self-reported OSPAQ indicated good or excellent test-retest reliability, as reflected by the ICCs for minutes spent sitting (0.66), standing (0.83) and walking (0.77) in Table 2. The paired sample t-tests also showed no significant differences between occasion 1 and occasion 2 across sitting, standing and walking domains.

**Table 2:** **OSPAQ (self-reported) measures administered at 7-day interval during the work day (n = 99)**

	Occasion 1		Occasion 2			Paired t-test		
Domain	Mean	SD	Mean	SD	***ICC***	Mean difference	t	p
					(95% CI)			
Sitting (min per week)	1917.9	471.6	1917.6	488.1	0.66 (0.49,0.77)	0.32	0.01	0.99
Standing (min per week)	149.7	232.7	148.4	158.6	0.83 (0.75, 0.89)	1.24	0.08	0.94
Walking (min per week)	177.6	137.2	180.5	128.5	0.77 (0.66, 0.85)	-2.91	-0.25	0.80

SD: standard deviation, CI: confidence interval, ICC: intraclass correlation coefficient.

### Criterion validity

Pearson (*r*) correlation coefficients between accelerometer-measured and self-reported intensity activities are shown in Table 3 for waist, thigh and combined locations. The results for minutes spent sitting over the course of the working week indicated strong associations with accelerometer data for time in sedentary activities for the waist location but low for the right thigh; for time spent standing the association was low to moderate for the waist location and moderate for the thigh location; whereas for time spent walking moderate associations for both the waist and thigh locations were evident. The validity for heavy labour items could not be determined due to the lack of data as such activities were not relevant to the office based environment.

**Figure 1:**
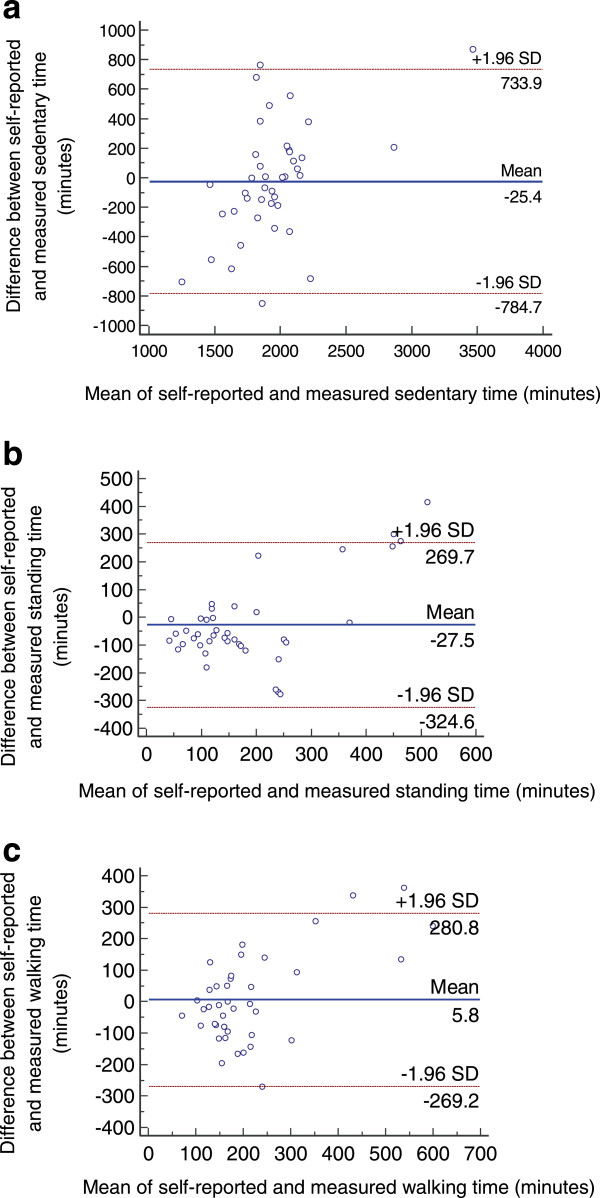
**Bland-Altman plots of discrepancy between self-reported and measured sedentary (a), standing (b) and walking (c) time versus the mean of self-reported and measured data.**

Figure 1 presents the Bland-Altman plots for sedentary time (1a), standing time (1b), and walking time (1c). Horizontal lines in each plot represent the mean discrepancy (solid line) and 95% limits of agreement (dashed lines). Systematic variations were observed for sedentary time, whereas standing time and walking time showed moderate agreement.

**Table 3:** **Criterion validity of OSPAQ when compared to accelerometer (five consecutive working days)**

OSPAQ	Accelerometer	Waist (n = 22)		Right thigh (n = 19)		Combined (n = 41)	
		***r***(95% CI)	***p***	***r***(95% CI)	***p***	***r***(95% CI)	***p***
Sitting	Sedentary	0.73 (0.45, 0.88)	<0.001	0.11 (-0.36, 0.54)	0.664	0.58 (0.33, 0.75)	<0.001
Standing	Light intensity	0.50 (0.10, 0.76)	0.019	0.61 (0.21, 0.83)	0.006	0.45 (0.17, 0.67)	0.003
Walking	Moderate intensity	0.55 (0.17, 0.79)	0.008	0.61 (0.21, 0.83)	0.006	0.45 (0.17, 0.67)	0.003

*r:* Pearson’s correlation coefficient, CI: confidence interval.

## Discussion

This study investigated the OSPAQ in terms of sitting, standing and walking time of office based workers during the working day. The results suggested that the self-reported instrument is acceptable for measuring sedentary and activity behaviours in the workplace, as evident from the good or excellent test-retest reliability with high observed ICCs for the three domains. It is short and easy to use, making it potentially suitable for applications to different target groups in a range of workplace settings.

The accelerometer was used to objectively measure the criterion validity of this instrument. Comparing self-reported physical activity data with an accelerometer has been recognised as a good practice for determining criterion validity with like variables [23]. The OSPAQ was found to be a valid instrument for quantifying sitting, standing and walking. Although the evidence remains inconclusive, our preliminary results suggest that the accelerometer data when combined have a strong association with the self-reported data. When the locations are separated (waist and thigh), the waist may be preferable than the thigh as the location to position the accelerometer because of its high association observed for sitting, while sitting accounted for 6.4 hours daily or 85% of their work day on average for these office based workers.

The Bland-Altman plots revealed mixed levels of agreement between methods (OSPAQ and accelerometer). The estimated limits of agreement suggest that a large proportion of this target group may over-report their sedentary time by as much as 734 minutes per week (approximately 12 hours) or under-report it by a similar amount. The Bland-Altman plots for standing time indicated that individual differences could vary by as much as 325 minutes per week (approximately 5.4 hours), while walking time showed the least amount of variation, with limits of agreement around ±275 minutes per week (approximately 4.6 hours).

Accelerometers are generally considered to be one of the better methods for providing evidence of validity for questionnaires measuring physical activity [22]. However, accelerometers are not the criterion standard measure for assessing sitting, standing and walking, though they are often used in the development of tools to measure sitting and physical activity [23]. There are a number of reasons for the variations. It may be due to the activity categories of accelerometer data compared with those of the self-reported data not being exact. For example, moderate intensity activity time was compared to self-reported walking time and light intensity activity time was compared to self-reported standing time. In addition, the determined cut-point for differentiating between activity levels (sedentary, light intensity, moderate intensity) for the accelerometer data, when compared to self-reported data, will impact on the results. Another reason is that the accelerometer inclination sensors might not be sufficiently sensitive. Activpal is an alternative device capable of measuring motion and determining posture/inclination [24], and potentially applicable in the office based environment, but it was deemed too expensive for the present study.

Several issues should be considered when interpreting the results. This study used a convenience sample of volunteers and participants were not randomly recruited from the institution. Therefore, selection bias could not be ruled out, especially since the majority of participants were university educated and female. Nevertheless, it should be acknowledged that more women than men are generally employed in office based roles. Moreover, only a subsample of the participants were assigned to stream two to wear the accelerometer for five work days due to resource constraints.

## Conclusion

The OSPAQ appears to have acceptable reliability and validity measurement properties in the office workplace setting. The preliminary results also suggest that attachment of the accelerometer to the waist may be preferable for office based workers to objectively measure their sedentary behaviour.

## Abbreviations

ICC: Intraclass correlations
OSPAQ: Occupational Sitting and Physical Activity Questionnaire.
